# Exploring caregiver challenges, digital health technologies, and healthcare support: a qualitative study

**DOI:** 10.3389/fdgth.2025.1587162

**Published:** 2025-06-06

**Authors:** Humairah Zainal, Xin Xiaohui, Julian Thumboo, Siang Joo Seah, Low Lian Leng, Fong Kok Yong

**Affiliations:** ^1^ Health Services Research Unit, Singapore General Hospital, Singapore, Singapore; ^2^ Department of Rheumatology and Immunology, Singapore General Hospital, Singapore, Singapore; ^3^ Duke-NUS Medical School, National University of Singapore, Singapore, Singapore; ^4^ Centre for Population Health Research and Implementation, Singapore Health Services Pte Ltd, Singapore, Singapore; ^5^ Department of Family Medicine and Continuing Care, Singapore General Hospital, Singapore, Singapore; ^6^ Division of Population Health and Integrated Care, Singapore General Hospital, Singapore, Singapore

**Keywords:** digital health, digital intervention, mobile application, caregiver, caregiving, caregiver skills training, qualitative research, Singapore

## Abstract

**Introduction:**

Informal caregiving often involves navigating complex healthcare tasks, a challenge that is amplified by the growing burden of chronic diseases and an ageing population. Digital health technologies (DHTs)—including mobile health apps, wearable devices, and telemedicine platforms—offer potential support, yet their integration into caregiving remains underexplored. This study investigates the challenges caregivers face when using DHTs in Singapore and identifies resources needed to optimize their use in everyday caregiving.

**Methods:**

Thirty informal caregivers of adults with chronic illnesses and/or physical or cognitive impairments were recruited through purposive sampling via caregiver and personal networks, and community organizations. Semi-structured interviews were conducted, and thematic analysis was used to identify key barriers and potential solutions.

**Results:**

Caregivers reported using DHTs such as the HealthHub app, Fitbit wearables, and video teleconsultation services. Seven key challenges emerged: (1) lack of formal training in DHT use, (2) difficulties providing timely care, (3) limitations of teleconsultations for complex needs, (4) poor app usability, (5) cost concerns, (6) age-related digital literacy gaps, and (7) cultural tensions in adopting DHTs. Solutions proposed included caregiver-targeted training programs, streamlined digital access to care, improved DHT design, equitable access to DHTs, age-inclusive healthcare services, affordability schemes, and culturally sensitive support.

**Discussion:**

This study highlights significant barriers to DHT adoption among informal caregivers and offers practical strategies to improve their use. Addressing these challenges through training, inclusive design, and equitable access can enhance caregiver resilience and system sustainability in digital health integration.

## 1 Introduction

Caregiving is a multifaceted and demanding role, presenting physical, emotional, and financial challenges (1). Caregivers provide crucial support to individuals with chronic health conditions or disabilities, navigating not only logistical burdens but also the need for social and healthcare support. The World Health Organization (WHO) defines a caregiver as someone who offers formal or informal support and assistance to individuals with disabilities, long-term health conditions, or those who are elderly (2). Formal caregivers are those who have undergone training and are paid for their services. Informal caregivers are unpaid and offer long-term care support for care recipients with whom they have a personal relationship (2). They provide care mostly to assist with personal tasks, including ADLs such as bathing, toileting, mobility, and continence, and IADLs like housekeeping, preparing meals, managing medication and using communication devices (2).

In response to the numerous challenges faced by caregivers, digital health technologies (DHTs) have emerged as promising tools to enhance caregiving practices. DHTs are defined as digital tools and systems that leverage information technology to support the management, diagnosis, and treatment of health conditions (3). These technologies—such as telemedicine platforms, mobile health applications (apps), and wearable devices—have been identified as valuable resources that assist caregivers in managing both daily caregiving tasks and complex clinical responsibilities (4–6).

The National Study of Caregiving (NSOC), a U.S. telephone survey of family and unpaid caregivers assisting participants in a longitudinal study of Medicare beneficiaries, categorizes caregiving tasks into three types: (1) those related to activities of daily living (ADL) and instrumental activities of daily living (IADL), (2) health management, and (3) health systems logistics (7, 8). ADL/IADL-related assistance includes help with transportation, housework, mobility, banking, shopping, and self-care. Health management involves support with diet, exercise, foot care, skin care, and dental care. Health systems logistics covers tasks such as scheduling appointments, managing medications, and communicating with healthcare providers (7, 8). While previous research has explored caregivers' experiences with DHTs, most studies have focused on how these technologies alleviate burdens related to ADL/IADL (3, 9, 10). For example, Ajay et al. (2017) primarily examine logistical assistance, including patient mobility, incontinence management, and home safety (10).

There are relatively fewer studies that have investigated how specific DHTs can be optimized to support caregivers in health systems logistics, despite their potential to improve care quality, patient outcomes, and coordination with healthcare stakeholders (5, 6, 11). Existing research offers valuable insights but often relies on quantitative methods that may not fully capture caregivers' complex needs (11, 12). For example, Moretta et al. (2024) examined the use of telemedicine in diagnosing disorders of consciousness. Similarly, Mishra et al.'s (2022) explored the Care4AD platform, which supports dementia caregiving through task scheduling and daily activity management using a smart tablet, mobile app, and wireless sensor tags (11).

There are three key research gaps this study seeks to address. First, there is a relative lack of qualitative studies that offer nuanced, in-depth insights into the challenges faced by informal caregivers. Second, existing qualitative research on caregivers' use of information and communication technology has largely been conducted in North America and Europe, with limited attention to the context-specific concerns of Asian caregivers. Third, most studies have focused on specific types of DHTs, offering limited understanding of the broader range of challenges caregivers face across various technologies. For instance, studies by Moretta et al. (2024), Mishra et al. (2022), and those included in Niazkhani et al.'s (2020) systematic review primarily focus on individual DHTs (5, 11, 13). To address these gaps, this study adopts a qualitative approach to explore the challenges informal caregivers in an Asian context encounter in adopting DHTs—including language barriers and culturally specific concerns—and the resources required for their effective use.

Singapore, a technologically advanced nation in Southeast Asia with strong government support for digitalization (14), offers a compelling case study to examine the challenges. The country's healthcare system integrates cutting-edge technologies like telemedicine, electronic health records (EHRs), and AI-driven solutions (14), making it an ideal location to study how caregivers interact with such tools. Despite these advancements, older populations, particularly those of lower income, face challenges with technology accessibility, usability, and digital literacy (14). Additionally, Singapore's multicultural society adds another dimension to DHT adoption. Among its citizen population, 75.6% are Chinese, 15.1% are Malays, 7.6% are Indians and 1.7% are from other ethnic groups (15). Given its multicultural population, caregivers from diverse linguistic and cultural backgrounds may encounter distinct challenges, such as language barriers and conflicting views on medical interventions. This research will explore how caregivers navigate the intersection of traditional caregiving practices with modern technologies, offering insights into inclusive, practical digital health solutions that align with diverse caregiving needs.

## 2 Materials and methods

### 2.1 Setting and sample

In this qualitative study, individual semi-structured interviews were conducted with informal caregivers, defined earlier as unpaid individuals who support people with chronic health conditions or disabilities, often through personal and household care activities. The inclusion criteria included those who were actively and primarily involved in at least two of the following duties: (i) providing direct care to care recipients for daily activities, (ii) actively participating in the decision-making for the care and treatment of care recipients, and/or (iii) supervising a migrant domestic worker in care duties (16). They could be either family members, friends, neighbors or other community members of those looking after patients with chronic health conditions and/or physical or cognitive impairment. They need not have any experience or knowledge with DHTs. Paid domestic helpers were excluded.

Participants were recruited using purposive sampling to capture diverse perspectives and select individuals with specific characteristics relevant to the study. Accordingly, data from caregivers looking after care recipients with varying medical conditions were collected to ensure broad representation of findings. Caregivers from different age categories, ethnic groups, and who lived in various types of housing were recruited regardless of their experience with DHTs to examine if their age, cultural and socio-economic background have any influence on their perceptions and use of DHTs. In particular, participants from different age bands, spanning between 21 and 40 years, 41 and 60 years, and above 60 years, were interviewed. Recruitment also targeted Singapore's majority and minority ethnic communities, namely, the Chinese, Malays, and Indians.

We recruited those living in both public and private housing. As housing type is closely linked to income level, it serves as a strong indicator of socio-economic background (17). Individuals residing in one- to two-room rental flats, and three-room public housing flats are typically classified as low-income, while those in four- or five-room flats are generally considered middle- or upper-middle-income. Participants living in maisonettes, executive apartments, executive condominiums, private condominiums, or landed properties are categorized as high-income. To contextualize participants' socioeconomic backgrounds, household income was classified into three broad categories based on national statistics from the Singapore Department of Statistics and publicly available government reports. While there are no official thresholds for defining low, middle, and high-income households, we used approximate monthly household income ranges. Low-income households were defined as those earning less than S$3,000 per month, corresponding to the bottom 20% of the income distribution (18). Middle-income households were those earning between S$3,000 and S$13,000 per month, covering the 21st to 80th income percentiles (18). High-income households were defined as those earning above S$13,000 per month, typically representing the top 20% of earners. These classifications were aligned with the 2024 median monthly household income in Singapore, which is S$11,297, and were used to guide the analysis of how socioeconomic status may influence caregivers' interaction with DHTs (18).

During recruitment, participants were briefly asked whether they had any prior experience with DHTs, such as health apps, teleconsultations, or wearable devices, to ensure variation in familiarity across the sample. These initial responses informed recruitment but did not determine eligibility. A more in-depth exploration of their engagement with DHTs was carried out during the interviews.

### 2.2 Ethics approval

This study was granted an ethical waiver by SingHealth Centralized Institutional Review Board (Reference Number: 2020/2880). This decision was based on the determination that the activities involved posed no more than minimal risk to participants. Despite this waiver, the research adhered strictly to the ethical principles outlined in the World Medical Association's Declaration of Helsinki and institutional guidelines.

### 2.3 Data collection

Data were collected from August to December 2024. We conducted four pilot interviews to evaluate the clarity, flow, and relevance of the interview guide. These pilot interviews helped us identify questions that required rephrasing for greater clarity and neutrality. For example, some questions were modified to be more open-ended and less leading. While we did not solicit formal feedback, we closely observed participants' reactions and responses, and used these insights to refine the wording and sequencing of certain questions. These initial interviews were included in the final analysis because they provided relevant data that directly addressed the research questions and contributed to insights significant to the study. We then carried out more interviews until data saturation was reached, ensuring that no new themes or insights emerged from the interviews.

Potential participants were identified by HZ through community centers, non-profit organizations, and personal networks, before receiving email invitations for the interview. The email detailed the study's purpose, procedures, potential risks, and benefits. It also included a consent statement for participants to review and acknowledge before proceeding. Interviews were then conducted and recorded over Zoom (Zoom Video Communications) unless participants requested an in-person interview. At the start of each interview, participants' verbal consent was recorded to ensure informed and voluntary participation. They were reminded of their right to withdraw at any time, with clarification that data collected before withdrawal would still be retained and analyzed for a comprehensive evaluation of findings.

The interview guide was developed based on themes from previous studies on caregivers' experiences with various DHTs in caregiving. We selected DHTs widely used by caregivers in published research and in Singapore, including healthcare apps, wearables, and telemedicine (3, 19–21). The questions were adapted to allow participants to share additional insights beyond the guide. Generally, questions explored participants' challenges with health systems logistics, experiences with DHTs, and how DHTs could address these challenges and improve caregiving support (Table 1).

**Table 1:** Interview questions.

Number	Questions
1.	• What challenges, if any, do you face when helping with healthcare-related tasks, like scheduling appointments or managing medications, for the person you care for?
2.	• How do you keep track of the care recipient's medical records and appointments, or treatment plans? Are there any technological tools, smartphone applications or health portal that you use for this purpose?
3.	• Are there any specific medical devices or wearables that you use to monitor the health and well-being of the care recipient? Examples of such devices include glucose monitoring device to measure glucose levels, and Fitbit watch to monitor heart rate.
	• *If yes:* How have these technologies improved the way you help the patient?
	• *If no:* Would you be open to using these technologies? Why or why not?
4.	• How do you communicate with the doctor who looks after your care recipient? Have you used any digital platforms when consulting the doctor? Examples include video consultation over Zoom, WhatsApp video calls or any other form of video consults.
	• *If yes*: What was the experience like & how do you feel about the quality of care when you had a consultation on such a platform?
	• *If no*: Would you be open to using these technologies? Why or why not?
5.	• How do you stay updated on the latest clinical research and treatment guidelines? For example, in looking for information on the treatment of your loved one's condition, do you use any technological tools or devices for this purpose?
6.	• What sources would you turn to if you had queries about the clinical aspects of caregiving between doctor consultations? How do you ensure they are legitimate?
	• Prompt: Are you open to using AI tools such as chatbots? Do you have any concerns about using these tools?
7.	• In your opinion, what kind of technologies hold the most promise for enhancing the healthcare support you provide to the care recipient?
	• Prompt: What are your thoughts on the use of robotics or assistive technology to help the care recipient with mobility or other issues he/she is facing?
8.	• What kind of support would help you effectively use digital technologies in your caregiving role?
	• Have you received any training on the use of digital technologies for healthcare-related tasks?
	• *If yes*, how has this training impacted your effectiveness in caregiving?
	• In what ways do you think you can be better supported when using digital technologies for caregiving?
9.	• Before we end this interview, do you have any other comments on the use of technology in caregiving, or the support you need in caregiving?

While the interview guide provided an initial framework for data collection, the analysis was conducted inductively to allow for the emergence of unanticipated themes. As such, the structure of the results section does not strictly mirror the sequence of the interview questions. Instead, themes were organized based on the patterns and meanings that surfaced most strongly across participants' narratives during coding. This approach allowed for a more nuanced representation of caregivers' experiences and perceptions, and ensured that the findings were grounded in the data rather than constrained by the interview structure. Table 4 reflects the final thematic framework developed during analysis, which guided the presentation of findings in the results section.

In reviewing reflexivity, we considered how our assumptions and positionality as researchers could influence the study (22). Caregiving is a sensitive topic, and the interviews often elicited personal and distressing information. Reflexivity required us to approach these moments with ethical responsibility and emotional sensitivity. Several participants became emotional during interviews, and we paused to offer them space, allowing breaks and prioritizing their well-being over data collection. Honoring the emotional weight of their stories helped ensure their voices were represented with respect and integrity.

Furthermore, the lead researcher identifies as a female academic with prior experience conducting qualitative interviews in health and caregiving contexts. While not a caregiver herself, her familiarity with healthcare systems and digital technologies may have influenced the interpretation of participants' experiences, especially in framing their challenges through a systems or innovation lens. To mitigate this, we adopted an open-ended interview approach and co-reviewed transcripts to ensure that emerging themes reflected participants' own narratives, rather than researcher expectations.

### 2.4 Data analysis

Thematic analysis using Braun and Clarke's six-step framework was employed to explore barriers that emerged from the data (23). This involved familiarizing ourselves with the data by reading the transcripts in their entirety before generating relevant codes, grouping them into themes and sub-themes, and defining the themes. To overcome potential interpretive bias and selective perception, coding was conducted by two researchers, FKY and HZ, independently. After initial independent coding of approximately 25% of the transcripts, the researchers compared and discussed discrepancies to reach consensus and refine the codebook. This iterative process helped ensure consistency in code application. Once consensus was achieved, the remaining transcripts were coded independently using the finalized codebook, with regular discussions held to resolve any emerging uncertainties.

To enhance the trustworthiness of the data, we conducted data triangulation by comparing findings across caregivers of different demographic and caregiving backgrounds, including variations in age, gender, ethnicity, and care contexts. This form of triangulation helped surface both common themes and divergent experiences (24). To protect participants' anonymity, we assigned code identifiers beginning with “CG” to each participant, an acronym for caregivers. In the reporting of findings, we followed O'Brien et al.'s (2014) Standards for Reporting Qualitative Research (Table 2) (25).

**Table 2:** Standards for reporting qualitative research.

Number	Topic	Item
Title and abstract		
S1	Title	• Exploring caregiver challenges, digital health technologies, and healthcare support: a qualitative study
S2	Abstract	• Please refer to the Abstract in the main text.
Introduction		
S3	Problem formulation	• The study aims to identify the challenges caregivers face when helping with healthcare-related tasks, and the resources needed to optimize the utilization of DHTs in caregiving.
S4	Research questions	• What challenges do caregivers face when undertaking healthcare-related tasks with DHTs?
		• What type of support and resources do caregivers need to optimize the utilization of DHTs in caregiving?
Methods		
S5	Qualitative approach and research paradigm	• This manuscript is based on a qualitative interview research with caregivers who are taking care of patients with chronic conditions and/or physical/cognitive impairment.
S6	Researcher characteristics and reflexivity	• Please refer to the Data Collection section for elaboration.
S7	Context	• This study uses Singapore as a case study. Singapore is a multi-cultural population with a high proliferation of digital technology and mobile phones, and where digital uptake is high among the population regardless of educational level (3, 19).
S8	Sampling strategy	• Participants were recruited using purposive sampling until data saturation was achieved.
S9	Ethical issues pertaining to human participants	• Waiver for ethical approval was granted by SingHealth Centralized Institutional Review Board (reference 2020/2880).
S10	Data collection methods	• Please refer to the Methods section in the main text for details.
S11	Data collection instruments and technologies	• Twenty-one interviews were conducted and recorded over Zoom, while 9 were done in person (upon participants’ request). The latter interviews were audio recorded. Each interview lasted approximately one hour.
S12	Units of study	• Thirty caregivers who were actively participating in the direct care, and/or decision-making of care recipients with chronic conditions, respiratory illnesses and/or physical or cognitive impairment took part in the one-off interview.
S13	Data processing	• The interviews were transcribed verbatim by Otter AI, a transcription software, and the transcripts reviewed by FKY and HZ to ensure transcription accuracy. To protect participants’ anonymity, we assigned code identifiers beginning with “CG” to each of them, an abbreviation for “caregivers.”
S14	Data analysis	• The researchers adopted an inductive thematic analysis approach when evaluating the data to draw common and shared meanings among participants. Coding frameworks and themes were developed iteratively using the six-step process proposed by Braun and Clarke (2006).
S15	Techniques to enhance trustworthiness	• Credibility: Data were collected from caregivers across diverse age groups, ethnicities, and socio-economic backgrounds to capture a range of perspectives and minimize bias.
		• Transferability: The findings were compared with relevant and up-to-date literature on caregivers’ experiences with and perceptions of DHTs, particularly in developed countries with similar healthcare digitalization trajectories to Singapore. This cross-contextual comparison supports the relevance and applicability of the findings.
Results and findings		
S16	Synthesis and interpretation	• Refer to the Findings section.
S17	Links to empirical data	• Refer to the illustrative quotes in the Results section. Full data are available upon reasonable request to authors.
Discussion		
S18	Integration with prior work, implications, transferability, and contributions to the field	• Refer to the Discussion section.
S19	Limitations	• Refer to the text under Strengths and Limitations.
Others		
S20	Conflicts of interest	• None was reported by the authors.
S21	Funding	• SingHealth Duke-NUS Medicine Academic Clinical Program under Seah Cheng Siang Distinguished Professorship in Medicine.

Twenty-one interviews were conducted over Zoom while 9 were held in-person based on the participants' preference. Each interview lasted approximately an hour and was audio-recorded. The transcriptions were derived from the audio recordings of the interviews, which were processed using Otter AI software before being reviewed for accuracy by FKY and HZ.

## 3 Results

A total of 30 caregivers participated in our study, representing diverse genders, ethnicities, socioeconomic backgrounds, educational levels and age groups. These caregivers were responsible for patients with various medical conditions including chronic ones such as diabetes, stroke, and asthma, with most being family members of the patients in their care. Among the participants, 12 (40%) belong to the low-income group, 9 (30%) are from the middle-income group, and another 9 (30%) are from the high-income group. Their demographics are detailed in Table 3.

**Table 3:** Demographics of participants (*N* = 30).

Characteristics	Participants, *n* (%)
Gender	
• Male	7 (23)
• Female	23 (77)
Age range (years)	
• 21–40	9 (30)
• 41–60	13 (43)
• >60	8 (27)
Ethnicity	
• Chinese	14 (47)
• Malay	12 (40)
• Indian	4 (13)
Educational qualification	
• Primary	1 (3)
• Secondary	7 (23)
• Post-secondary: A level/Diploma/ITE/other post-secondary qualification	8 (27)
• University degree: Bachelor's, Master's, Ph.D.	14 (47)
Housing type	
• Rental flat: 1 or 2 room	4 (13)
• 3 room	8 (27)
• 4 or 5 room	9 (30)
• Maisonette/executive condominium/executive apartment	4 (13)
• Private condominium/landed property	5 (17)
Duration of caregiving for existing care recipient (years)	
• <1	2 (6)
• 1–5	11 (37)
• 6–10	12 (40)
• 11–15	5 (17)
Main care services sought by care recipient (Note: Some patients rely on a combination of primary healthcare services to address their healthcare needs)	
• General practitioner (private clinic)	4 (13)
• Homecare	2 (7)
• Public primary healthcare clinic (known as “polyclinic” in Singapore)	10 (33)
• Public hospital (specialist care)	20 (67)
• Private hospital (specialist care)	1 (3)
• Others (e.g., home nursing care)	2 (7)
Primary medical condition of care recipient (Note: Most patients have multimorbidity)	
• Alzheimer's disease	2 (7)
• Asthma	1 (3)
• Autism	2 (7)
• Crohn's disease	1 (3)
• Breast cancer	1 (3)
• Dementia	8 (27)
• Diabetes	8 (27)
• Down's Syndrome	1 (3)
• Epilepsy	1 (3)
• Heart failure	3 (10)
• Hypertension	6 (20)
• Hyperthyroidism	3 (10)
• Liposarcoma	1 (3)
• Kidney failure	3 (10)
• Parkinson's disease	2 (7)
• Pneumonia	2 (7)
• Spinal Cerebellar Ataxia	2 (7)
• Stroke	5 (17)

Among them, 21 (70%) had used a healthcare app in their caregiving duties, 17 (56.7%) had used a medical monitoring device, and 10 (33.3%) had experience with teleconsultations with the patient's primary doctor. Five participants (16.7%)—three of whom had either primary or secondary education- had no experience with any of the DHTs. Overall, participants expressed openness to adopting new DHTs that showed potential benefits for patients.

Participants mentioned seven key challenges when managing healthcare-related tasks, which are explained below. The analysis of codes, along with the generation of subthemes and themes, is summarized in Table 4.

**Table 4:** Codes, sub-themes and themes identified from coding process.

Codes	Sub-themes	Themes
Lack of training in using DHTs No experience/Lack of experience in using DHTs New challenges Interpreting results Reliance on informal learning	Caregiving knowledge and skills gaps Inadequate structured training opportunities Reliance on self-learning or guidance from peers and family members	Lack of formal training in DHTs
Anxiety Long waiting time Not available Not enough support Panic Tedious process	Lack of immediate access to emergency services Limited availability of healthcare professionals Inadequate and complex emergency response systems	Difficulty in getting prompt healthcare access and professional support
Brief, limited interaction Lack of detailed instructions Prefer human touch Expectations: Show empathy, do a thorough check Poor quality of call	Challenges in building trust and rapport with healthcare providers Difficulty diagnosing complex conditions remotely Patient reluctance or discomfort with virtual platforms Technological issues affecting consultation quality	Limitations of teleconsultation for complex health conditions
Cannot view medical results Challenge in rescheduling appointments Complex user interfaces Inconvenient Interpreting results Lack of intuitive navigation Low uptake Multiple apps Phone call hassle	Insufficient user guidance and support Lack of interoperability Navigation and interface design challenges Standardized process needed to engage data meaningfully	Usability and functionality challenges
Expensive High cost Limited government subsidies	Economic disparities in access to DHTs High cost of DHTs Limited availability of subsidies	Affordability issues in using certain DHTs
Dismissive Hurtful Old already Selective Too many patients, just one machine	Bias in prioritizing younger patients for technology-based care Bias in resource allocation and care delivery Exclusion from digital healthcare innovations Healthcare providers’ attitudes and behavioral biases Lack of age-friendly technology designs	Perceived age-related barriers to access healthcare technologies and services
Cultural values emphasizing familial caregiving roles Family support Sibling support	Cultural preference for traditional caregiving approaches Reliance on informal caregiving networks Perception of digital tools as supplementary rather than primary care solutions	Balancing traditional caregiving with DHT benefits

### 3.1 Lack of formal training in DHTs

One common challenge shared by the majority of participants, regardless of age, income and educational levels, was the lack of formal training in using DHTs. Even younger participants, like CG6, who considered themselves technologically savvy, acknowledged the need for structured training to effectively use DHTs and manage their loved ones’ health conditions. CG6, for example, expressed frustration over her limited knowledge in interpreting readings from monitoring devices for her mother's atrial fibrillation (AFib), which often resulted in unnecessary emergency calls.

There are other things I need to know too, like how to use monitoring devices, especially since my mom has AFib. I want to know what readings are normal and what aren't. Sometimes her heart rate fluctuates, and I'm not sure of what to do. Without proper training or guidance, I often end up calling an ambulance when I see signs of AFib. It's frustrating because this kind of training is not readily available. (CG6, 36)

Similarly, another caregiver who is in her 30s and is university-educated, CG28, opined that training in performing first aid on the patient under her care, especially when DHTs are required, would be very helpful.

Caregivers should know how to perform first aid, including Cardiopulmonary Resuscitation (CPR), and use devices like the Automated External Defibrillator (AED), which uses sensors and algorithms to assess heart rhythm and determine if a shock is needed to restore a normal heartbeat. For example, how do you perform it on patients who are on a wheelchair? Or, what if they are choking? I do not have all this information at hand. I have to learn it online. So if there is training on this, it would so beneficial. (CG28, 35)

While tracking devices and sensors hold great potential for supporting patients with conditions like Down's Syndrome, there is limited proper training in using these tools, as emphasized by CG7 below. Furthermore, time constraints, due to caregiving duties and work commitments, may hinder caregivers from attending relevant workshops.

A tracking device could help monitor her whereabouts, especially since she has gone missing before and was only found by a police officer later that day at a bus stop in Bedok, without any identification on her. A sensor to track her movements at home would also be useful, particularly as she frequently goes to the toilet at night. But I don't know how to use them. I don't have the time to attend training sessions either. (CG7, 58)

### 3.2 Limitations of DHTs in providing real-time support

Barriers in healthcare support, such as the lack of timely responses from healthcare providers and immediate access to teleconsultation, significantly affect caregivers' attitudes towards technology. While DHTs have the potential to bridge these gaps, caregivers continue to face challenges with their accessibility, usability, and integration into healthcare systems, leaving them struggling to address urgent medical concerns efficiently. For example, one caregiver described how delayed communication with the hospital made it difficult to manage her mother's medication, despite the availability of digital tools:

Administering medication to my mother has been challenging, as she struggles to swallow tablets and often spits them out. Although her doctor switched her prescription to a liquid form, it wasn't suitable for her either. When I tried to reach out to the hospital to speak with the doctor about finding a better solution, it took several days for the doctor to respond, making it difficult to address her needs promptly… I resorted to using a chatbot, but the answers were very generic and not helpful. (CG5, 69)

Another caregiver expressed frustration over the limited availability of immediate support from community nurses, particularly during emergencies, and highlighted the shortcomings of existing digital healthcare solutions:

The health app provided only general advice, which wasn't helpful in an urgent situation. I couldn't reach the community nurse immediately, and the limited support during an emergency was very stressful. I wish there was a faster teleconsult option. (CG10, 35)

These examples illustrate not only the lack of timely communication with healthcare professionals but also the limitations of current DHT solutions. Digital platforms, such as chatbots and healthcare apps, often provide limited medical information and advice. Teleconsultation services, while already available in some public healthcare institutions and clinics, are not accessible to those who need urgent medical assistance. This underscores the need for more responsive, accessible, and well-integrated digital healthcare solutions.

### 3.3 Limitations of teleconsultation for complex health conditions

The participants also emphasized the need for more comprehensive support through teleconsultations, as they frequently rely on these sessions for guidance in managing their care responsibilities. Many appreciated the convenience of teleconsultations, noting that they save time and money for routine concerns. However, those caring for patients with complex conditions, such as spinal cord injuries and dementia, expressed concerns about the lack of thoroughness compared to face-to-face visits, particularly when detailed examinations or physical assessments were necessary. For instance, some caregivers found teleconsultations too brief or lacking depth:

I prefer face-to-face consultations, as I feel more satisfied when the doctor performs a thorough check, including a physical examination, which isn't possible over Zoom. Teleconsultations feel too brief, and I expect a more comprehensive assessment.” (CG23, 38)

These experiences underscore caregivers’ need for clearer and more detailed instructions and assurance during teleconsultations. Without in-person interactions and physical assessments, they may struggle to obtain the necessary guidance to confidently manage their loved ones' conditions.

### 3.4 Usability and functionality challenges

Many caregivers relied on healthcare apps to access medical records and test results. However, usability and functionality challenges remained. CG12 valued the ability to review the lab test results of her mother through HealthHub but found the lack of access to other reports inconvenient, suggesting a more comprehensive approach.

It's good that I can view the lab test results on HealthHub, but why can't I access x-ray or ultrasound results through the app as well? Every time I need those reports, I have to pay for them unless I ask the doctor in person. It would be much more convenient if the app provided a more comprehensive view of all test results. Another issue is with appointment scheduling. The app restricts users from rescheduling appointments, which is frustrating. Calling the hotline takes too long, and I end up wasting my mobile minutes. It would be much more user-friendly if I could manage everything directly through the app. (CG12, 54)

Similarly, CG15 found the apps useful but felt that having multiple platforms added unnecessary complexity:

HealthHub and HealthBuddy both offer useful features, but why do we need two separate apps? It would make more sense to combine all functions into one platform to simplify access. (CG15, 58)

These insights highlight the benefits of healthcare apps in providing digital access to medical records and appointments while underscoring the need for improved integration and functionality to enhance user experience.

### 3.5 Affordability issues in using certain DHTs

The cost of certain DHTs poses a barrier for some caregivers who may find them beneficial but unaffordable. For example, CG28, a young caregiver who comes from a low-income family and is caring for her mother who had diabetes, began purchasing a continuous glucose monitoring sensor only after securing full-time employment. She chose this method to obtain her mother's glucose readings despite the sensor's perceived high cost, finding it less invasive than the traditional finger-pricking method.

My mum used to prick her fingers four times a day for years, which was very uncomfortable for her. When this new technology was introduced, I was initially hesitant due to the high cost. The sensor is quite expensive, lasting only 14 days, which means two sensors are needed per month, amounting to $180. It took me about five years after its introduction to decide to purchase it for her. However, it has proven to be much less painful and invasive, which greatly improved her experience. (CG28, 35)

For caregivers from high socio-economic background, the cost of glucose monitoring sensors is not a significant issue. In fact, the experience of monitoring glucose levels is often part of a comprehensive care package. For instance, some caregivers such as CG2, use advanced systems like the Libre sensor, which connects to an app for real-time glucose monitoring. They may scan the sensor and maintain direct communication with healthcare providers through platforms like WhatsApp. In such cases, adjustments to insulin dosages are managed promptly, and regular doctor consultations—whether through home visits or video calls—are included in a chronic diabetes management package. Additional devices, such as blood pressure monitors, are also integrated into daily care routines.

For daily care, my mother-in-law uses a Libre sensor connected to an app, scanning three to four times a day to monitor her glucose levels. We stay in touch with the doctor via WhatsApp to report any significant changes in her sugar levels, and insulin dosages are adjusted as needed. Each morning, we also check her blood pressure, both lying down and sitting up. We're on a chronic diabetes management package, which includes occasional home visits from the doctor and regular video consultations to ensure everything is in order. (CG2, 54).

Thus, while the cost of DHTs, such as glucose monitoring sensors, presents a significant barrier for some low-income caregivers, wealthier ones often access these tools through comprehensive care packages. This contrast highlights the disparities in access to advanced healthcare technologies.

### 3.6 Perceived age-related barriers in accessing healthcare technologies and services

Caregivers also face challenges in navigating health systems logistics when DHTs are limited or selectively allocated. One caregiver, CG16, shared her experience supporting her husband's rehabilitation, which involved a robotic-assisted device known as the Lokomat. While the technology had significantly improved his mobility, access was severely limited- there was only one machine available for a large number of patients, with older adults sometimes dismissed by healthcare providers as “too old” to benefit.

The Lokomat is helpful but access is limited with only one machine available for many patients. Unfortunately, older patients are sometimes dismissed by doctors and physiotherapists, who claim the equipment won't benefit them due to their age, which feels very hurtful. (CG16, 63)

Another caregiver, CG21, shared how her grandmother's health concerns were often minimized at polyclinics, with staff attributing issues to age rather than addressing them seriously. She was apprehensive that this neglect might be exacerbated in teleconsultations, where the absence of in-person interaction might further hinder elderly patients from having their concerns acknowledged.

My grandmother values in-person consultations, appreciating the human touch. But she often feels dismissed at polyclinics when sharing concerns about allergies or pain, with responses like, “You’re old, just rest more.” For her, issues like persistent itchiness are serious, but they aren't always taken seriously. It seems like elderly patients are often overlooked. This kind of discrimination could be further amplified in teleconsultations, where the lack of face-to-face interaction may make it even harder for elderly patients to convey their concerns and feel heard. (CG21, 25)

Both examples illustrate how age-based biases can leave elderly patients feeling dismissed and undervalued in their healthcare experiences. They also highlight caregivers' concerns that DHTs may not be inclusive enough, as they risk overlooking the specific needs of elderly patients who already struggle to have their concerns taken seriously in traditional healthcare settings. The challenge lies in ensuring that DHTs such as telemedicine platforms provide the same level of attentiveness and empathy as in-person visits, rather than further marginalizing elderly patients.

### 3.7 Balancing traditional caregiving with DHT benefits

Many participants from the Malay community expressed concerns about balancing traditional family caregiving expectations with the potential benefits of using DHTs. While all Malay participants recognized the usefulness of technology, they emphasized that caregiving is deeply intertwined with cultural and moral values that prioritize strong familial ties, communal support, and a sense of duty to care for family members. For some, such as CG19 and CG20, the impersonal nature of technology was seen as inadequate for addressing the emotional and relational aspects that are central to caregiving, creating tension between embracing innovation and upholding deeply rooted family responsibilities. While caregiving is valued across other ethnic communities, this concern is particularly prominent within the context of the Malay cultural outlook on caregiving.

It's not that we don't want to use technology, but my cousins, siblings, and I believe that family support is very important, even more important than technology. Since our grandmother cared for us when we were young, it feels only natural for us to return that care. This is why we are hesitant to rely on technology—if one of us is unable to care for her, another family member will step in. We take comfort in knowing she is looked after by those who love her. This deep-rooted cultural commitment to caring for family is something I hope to pass down to my children. (CG19, 42)

My mother gets stressed easily, so my siblings and I take on the responsibility of caring for her to make sure she has all the support she needs. This allows her to focus on taking care of my grandmother. In our family, we believe that family support is the most important thing… I'm open to using technology in caregiving especially if it's helpful for us. But we also don't want to rely too much on technology- it's the human connection that matters most. (CG20, 35)

These perspectives highlight the centrality of family support in caregiving within the Malay community, where caregiving is viewed not just as a responsibility but as a moral obligation rooted in cultural values. While technology offers practical benefits, its perceived inability to address the emotional and relational dimensions of care underscores the importance of culturally sensitive approaches to integrating DHTs into caregiving practices.

## 4 Discussion

### 4.1 Principal findings

The findings highlight that the participants encountered multiple challenges when assisting care recipients with healthcare-related tasks. Seven common challenges involving DHTs emerged: lack of formal training, difficulties in providing timely caregiving support, limitations of teleconsultations for complex conditions, usability and functionality issues with healthcare apps, affordability concerns, perceived age-related barriers, and tensions between cultural expectations and the benefits of DHT use. While age, socio-economic background, education, and culture did not significantly affect caregivers' technological competencies, these factors influenced the type and extent of support they required.

Despite these challenges, DHTs offer significant benefits by enhancing healthcare accessibility, efficiency, and patient outcomes. Teleconsultations reduce travel burdens, allowing patients—especially those with mobility challenges or chronic conditions—to receive timely medical advice from home. This not only saves time and costs but also minimizes exposure to infectious diseases. Wearable devices enable real-time health monitoring, facilitating early detection of potential issues, while mobile health apps provide caregivers with easy access to medical records and appointments, streamlining patient management. Integrating these technologies improves care coordination, and optimizes resource allocation.

Unlike previous studies that link demographic factors to digital competencies and DHT use (12, 26, 27), our findings present a more nuanced perspective. Rather than determining competency, factors such as age and socio-economic status shape the level of assistance needed to integrate DHTs into caregiving. In particular, age-related barriers influence perceptions of value and access to resources, with older adults often facing devaluation in healthcare practices. While the findings foreground age and class, it is worth noting that other groups, including women, also face discrimination in healthcare systems, which can similarly affect their engagement with digital technologies. This underscores the need for tailored support that aligns with caregivers' specific contexts and challenges.

Furthermore, contrary to studies that frame technology adoption as a skill-based issue (12, 26, 27), this research highlights broader contextual barriers. It emphasizes that effective DHT use depends not only on individual competencies but also on infrastructure and user-centered design. Caregivers' frustrations with teleconsultations and apps reveal the need for better-designed systems rather than training alone. Moreover, by linking technological adoption to structural and cultural factors, this study shifts the focus from competency to advocating for tailored support and responsive healthcare systems**.** While governments promote digital adoption, some seniors still struggle with digital tools, relying on caregivers and adding to their burden. Overemphasizing digital solutions without alternative options risks excluding those unable to keep up. This underscores the need for holistic interventions that enhance caregivers' digital literacy while addressing service gaps in clinical care.

In Singapore, several government and institutional initiatives support caregivers in adopting DHTs. For example, the *Caregiver Support Action Plan*, spearheaded by the Ministry of Health, includes efforts to strengthen caregiver training and access to resources, some of which involve digital platforms. The Agency for Integrated Care (AIC) also offers digital tools and training workshops to help caregivers manage patients' health at home, such as through tele-rehabilitation and remote monitoring services. These efforts aim to improve digital literacy, reduce caregiver burden, and ensure continuity of care. Such models may offer valuable insights for other countries, particularly those grappling with similar demographic shifts. By embedding caregiver support into national digital health strategies and emphasizing user-friendly technologies, other health systems can better integrate DHTs into long-term care practices.

Unlike past studies that associate low income with low digital literacy at the outset of the research (28, 29), our findings show that caregivers from lower-income households and who do not possess a high educational qualification are also technologically savvy. Singapore's English-speaking environment enables even those with limited education to engage with digital technologies, as most apps and portals are in English. Additionally, widespread smartphone and internet access ensures that caregivers, regardless of income level, have constant exposure to digital technologies (3, 30). This regular interaction with technology helps caregivers build practical digital skills, enabling them to at least utilize healthcare apps and platforms. This has global implications, highlighting the need for policies that ensure equitable digital access, particularly in rural and low-income communities.

Many challenges identified in this study align with previous research on medical and caregiving app usability. Bendixen et al. (2017) found similar barriers, including frequent app updates and difficulty accessing critical medication information such as dosages, side effects, and interactions (31). Lobo et al.'s (2023) review of 47 mHealth apps for stroke caregiving engagement also reveals inadequate pre-release evaluation and a lack of usability-focused design (32, 33). These findings highlight the need for comprehensive, well-designed apps that effectively support caregivers and improve patient outcomes.

To ensure effective and inclusive DHTs, it is crucial to consider the complex interplay between technology, health, and culture. Cultural attitudes and values shape how different groups engage with technology. Nittas et al. (2024) emphasize the importance of consulting a wide range of stakeholders, including community leaders and the target audience, to ensure that technological interventions align with cultural expectations (34). Tran et al.'s study also shows how familism shapes caregiving in Latino and Asian American communities in the U.S., leading to distinct coping styles in caregiving (35). Given these variations, culturally tailored DHT programs are essential to address diverse caregiving experiences.

### 4.2 Recommendations

#### 4.2.1 Enhance caregiver support through accessible training programs

To address the challenge of effectively utilizing DHTs, structured and accessible training that covers essential caregiving skills and device usage like interpreting readings and performing first aid, are crucial for caregivers. Formal training, especially for complex conditions, can empower caregivers, reduce unnecessary emergency interventions, and enhance care quality.

Improving accessibility is key, as many participants struggled to attend in-person sessions due to work and caregiving demands. Virtual and community-based training options can ease this burden. Research shows well-implemented and virtual education can boost caregiver confidence as effectively as in-person programs (36).

Additionally, ongoing support, such as interactive technologies and regular follow-ups, enhances caregiver preparedness, though this requires sustainable implementation (37). While caregivers value personalized guidance, nurse workloads must be considered (37). Utilizing trained clinicians or caregiver support specialists can ensure tailored training without overburdening nurses (36).

#### 4.2.2 Enhance timely access to healthcare support through digital platforms

Timely access to healthcare support is essential for caregivers. Digital platforms such as teleconsultations and wearable technologies, can play a critical role in achieving this. To strengthen service delivery, healthcare systems should expand telehealth services and improve the interoperability between digital tools and EHRs. This will enable real-time information sharing, reduce conflicting advice, and enhance continuity of care.

Clear and responsive communication between caregivers and healthcare professionals, particularly during critical moments, also needs improvement. Seamless digital communication can alleviate caregiver stress and improve care outcomes. However, current challenges such as fragmented systems, server issues, and insufficient equipment point to the need for a robust digital infrastructure (38).

Wearable technologies offer promising solutions. For example, Jiang et al. (2023) demonstrated the use of a medical-grade smartwatch with real-time monitoring and cloud-based alerts to support stroke patients in China (39). Future research should explore how similar technologies can provide caregivers with timely, personalized alerts, medication reminders, and actionable insights to support care delivery.

#### 4.2.3 Optimize telemedicine for comprehensive care in complex cases

To address the limitations of telemedicine for complex cases, healthcare professionals need specialised training to optimise video-based assessments and guide caregivers in basic physical checks. While full physical examinations remain a challenge, Benziger et al. (2020) highlight how patient-assisted virtual examinations, combined with wearables and home-based tools like pulse oximeters, can enhance the accuracy of remote diagnoses (40).

Beyond clinician training, robust feedback systems are essential for refining telemedicine protocols. Caregivers and patients should have structured avenues to share experiences, enabling continuous refinement of telemedicine protocols and ensuring virtual care remains responsive to complex needs.

Moretta et al. (2024) emphasize caregiver involvement in telemedicine, particularly for disorders of consciousness (DOC). Their study found that caregivers providing auditory stimulation during Coma Recovery Scale-Revised (CRS-R) assessments improved diagnostic accuracy, leading to better detection of residual verbal abilities and more tailored treatment plans.

These findings underscore the value of caregiver integration in telemedicine, preventing care disruptions and enhancing rehabilitation continuity. Establishing protocols for clinician follow-ups and home-based care can further improve long-term outcomes for complex medical cases.

#### 4.2.4 Improve healthcare app usability and accessibility

To enhance healthcare apps' usability and effectiveness, a user-centered design approach involving key stakeholders is essential. Ha et al. (2023) emphasize partnering with individuals with disabilities, caregivers, and healthcare professionals from the design phase to integrate clinical expertise and promote digital health equity (41). Similarly, Bendixen et al. (2017) highlight the value of engaging end users through focus groups and surveys to refine app features and align them with clinical guidelines (31).

Our findings suggest key improvements: consolidating test results (e.g., x-rays, ultrasounds) within a single app, enabling direct appointment rescheduling, unifying multiple healthcare apps, and providing multilingual instructions. These enhancements would streamline navigation, boost adoption, and improve DHTs' overall effectiveness.

#### 4.2.5 Ensure equitable access to essential DHTs for all caregivers

Ensuring equitable access to DHTs requires inclusive design and implementation. Essential tools like glucose monitoring sensors should be universally available to all caregivers of diabetes patients, regardless of socio-economic status. Providing information on subsidies and financial aid can further reduce financial barriers and improve access.

Crawford and Serhal's (2020) Digital Health Equity Framework highlights that equity extends beyond access to ensuring improved health outcomes (42). Advancing digital health equity requires the incorporation of equity-oriented data in research, along with measuring access and outcomes across diverse populations (42). Actively involving communities in design, evaluation, and policy-making, along with diverse representation in leadership, ensures DHTs effectively serve all users.

#### 4.2.6 Foster age-inclusive healthcare access and support

To address age-related barriers in healthcare, policies should ensure equitable access to DHTs and treatments, allocating resources based on need rather than age. Expanding age-appropriate technologies and training providers to assess eligibility for assistive devices based on clinical evidence, not assumptions, is essential.

With teleconsultations increasing, elderly patients need adequate technical support, and their concerns should not be dismissed as age-related. Telehealth protocols should encourage active engagement with older patients and caregivers, ensuring their voices are heard.

#### 4.2.7 Bridge cultural values and DHTs in family caregiving

To ease cultural tensions in caregiving and technology adoption, educational programs should show how DHTs complement rather than replace traditional caregiving values. Demonstrating how technology enhances family care, such as improving health monitoring while maintaining relational bonds, can help bridge this gap.

Partnering with community leaders, religious scholars, and elders to co-develop and endorse DHTs through outreach can provide culturally relevant guidance and ease concerns about diminishing familial roles. Training healthcare professionals in cultural competence further ensures caregivers feel supported in ways that align with their values (43). Additionally, DHTs should facilitate shared caregiving, such as apps that allow multiple family members to monitor health or coordinate caregiving tasks collaboratively.

### 4.3 Strengths of the study

This qualitative study examines caregivers’ challenges in managing healthcare logistics and the support needed to optimise DHT use. By interviewing caregivers from diverse backgrounds, it highlights how contextual factors shape their experiences and reveals gaps in existing health systems, such as insufficient services and resources. The findings can inform personalized healthcare strategies and guide DHT design and integration by identifying key adoption barriers and facilitators.

### 4.4 Limitations of the study

A key limitation is the small sample size, which, while achieving thematic saturation, may limit the diversity of perspectives captured. However, the in-depth analysis provides valuable insights for digital health initiatives, particularly in multicultural and ageing populations. Another limitation is the lack of language diversity, as most participants were English-speaking, potentially excluding cultural and class-based perspectives. Despite this, participants varied in ethnicity, education, and socio-economic status.

Additionally, differences in digital literacy influenced participants' views on DHTs, with some struggling to assess future technologies. The study focused on DHTs in Singapore's healthcare system, and future research should explore broader technological innovations for caregiving support.

## 5 Conclusions

In conclusion, this study highlights the challenges informal caregivers face in healthcare-related tasks while integrating DHTs into their caregiving practices. By examining the experiences of caregivers in Singapore, the findings reveal significant barriers, including inadequate training, usability issues, as well as affordability and accessibility concerns. Addressing these gaps requires targeted solutions such as structured caregiver training, improved app design, equitable access, and age-inclusive healthcare services. These insights underscore the importance of developing inclusive, user-centered strategies to enhance DHT adoption and utilisation. By fostering greater caregiver resilience and improving care outcomes, this research contributes to advancing sustainable, digitally driven caregiving frameworks that align with evolving healthcare demands in ageing societies.

## Data Availability

The raw data supporting the conclusions of this article will be made available by the authors, upon reasonable request.
